# The prevalence of pediatric metabolic syndrome—a critical look on the discrepancies between definitions and its clinical importance

**DOI:** 10.1038/s41366-020-00713-1

**Published:** 2020-11-18

**Authors:** Carolin Reisinger, Benedicta N. Nkeh-Chungag, Per Morten Fredriksen, Nandu Goswami

**Affiliations:** 1 Physiology Division, Otto Loewi Research Center, Medical University of Graz, Graz, Austria; 2 Walter Sisulu University, Mthatha, 5117 South Africa; 3 School of Health Sciences, Kristiania University College, Oslo, Norway

**Keywords:** Risk factors, Metabolic syndrome, Metabolic syndrome

## Abstract

**Introduction:**

The Metabolic Syndrome (MetS) describes the clustering of cardio-metabolic risk factors—including abdominal obesity, insulin resistance, elevated blood pressure, high levels of triglycerides, and low levels of high-density lipoproteins—that increase the risk for developing cardiovascular diseases and type 2 diabetes mellitus. However, a generally accepted definition of MetS in pediatric patients is still lacking.

**Objectives:**

The aim was to summarize current prevalence data of childhood MetS as well as to discuss the continuing disagreement between different pediatric definitions and the clinical importance of such diagnosis.

**Methodology:**

A systematic literature search on the prevalence of pediatric MetS was conducted. Articles that were published during the past 5 years (2014–2019), using at least one of four predetermined classifications (International Diabetes Federation, Cook et al., Ford et al., and de Ferranti et al.), were included.

**Results:**

The search resulted in 1167 articles, of which 31 publications met all inclusion criteria.

**Discussion:**

The prevalence of MetS ranged between 0.3 and 26.4%, whereby the rising number of children and adolescents with MetS partly depended on the definition used. The IDF definition generally provided the lowest prevalences (0.3–9.5%), whereas the classification of de Ferranti et al. yielded the highest (4.0–26.4%). In order to develop a more valid definition, further research on long-term consequences of childhood risk factors such as abdominal obesity, insulin resistance, hypertension, and dyslipidemia is needed. There is also a temptation to suggest one valid, globally accepted definition of metabolic syndrome for pediatric populations but we believe that it is more appropriate to suggest definitions of MetS that are specific to males vs. females, as well as being specific to race/ethnicity or geographic region. Finally, while this notion of definitions of MetS specific to certain subgroups is important, it still needs to be tested in future research.

## Introduction

During the past decades, the world population has undergone significant changes in health and eating behavior and lifestyle [1–3]. These changes are seen in the increasing consumption of high-calorie food and sugary beverages as well as more sedentary behavior and severe lack of physical exercise [2, 4, 5]. As a consequence, the global prevalence of overweight and obesity has continuously been growing and has now reached epidemic proportions [6–10]. In parallel with this development, the prevalence of obesity-associated health consequences like cardiovascular diseases (CVDs) and diabetes mellitus type 2 (T2DM) has been increasing as well [11–14]. The metabolic syndrome (MetS) is frequently used to describe the pathophysiological connection between these trends. MetS is described as a combination of cardio-metabolic risk factors that are known to predispose an individual to CVD and T2DM. These components include central obesity, characterized by high-waist circumference (WC), dysglycemia/insulin resistance (IR), hypertension, high levels of triglycerides (TG), and low levels of high-density lipoproteins (HDL) [15]. The pathogenesis of MetS is complex and up until now, many aspects are still not fully understood [12, 15–18]. It is believed that central obesity and/or IR initiate many different pathogenic pathways that increase metabolic risk and end up in the full expression of the syndrome [15, 19]. Regarding the growing number of childhood MetS, cardio-metabolic abnormalities and MetS are expected to become more prevalent in youth as well [2, 20–22]. The early onset of risk factor clustering is alarming, considering that MetS components may track into adulthood and significantly increase the risk for future T2DM and CVD [23].

However, identifying those who are affected is rather difficult because clear recommendations about how to diagnose MetS in the young age group are still lacking [23]. Since study groups usually developed their own unique set of diagnostic criteria, we are now faced with a high number of different pediatric MetS classifications, each of which promotes its own limit values and measurement techniques [12]. In a literature review published in 2007, Ford et al. identified as many as 46 different definitions being used for assessing MetS in children and adolescents [24]. Most of them are based on the MetS classifications for adults [12] with one of the most prominent templates coming from the National Cholesterol Education Program Adult Treatment Panel III (NCEP-ATPIII) [25]. It is important to recognize that adult definitions of MetS cannot be applied to children, as classification of MetS in children requires the usage of age- and sex-specific percentiles and pediatric thresholds. Taking these aspects into account, Cook et al. [6], Ford et al. [26], and de Ferranti et al. [27] modified the NCEP criteria by replacing the adult cutoffs with age- and sex-specific percentiles and pediatric thresholds (Table 1) [22]. In 2007, the International Diabetes Federation (IDF) proposed a new set of diagnostic criteria with the aim to offer “a simple easy-to-apply definition” for clinical practice (Table 1) [28].

**Table 1:** Different definitions of pediatric metabolic syndrome, proposed by the IDF [25], Cook et al. [6], Ford et al. [26], and de Ferranti et al. [27].

	Abdominal obesity	Hypertension	Dyslipidemia	Fasting glucose
IDF [25] Central obesity + 2 of 4^a^	10–15 years of age WC ≥ 90th percentile	Systolic BP ≥ 130 mmHg	TG	≥100 mg/dl
			≥150 mg/dl	or diagnosis of type 2 diabetes mellitus
	>15 years of age WC ≥94 cm (♂)^b^ WC ≥ 80 cm (♀)^b^	or diastolic BP ≥ 85 mmHg	or specific treatment	
			HDL < 40 mg/dl (♂) <50 mg/dl (♀)	
		or specific treatment		
Cook et al. [6] 3 out of 5^a^	WC ≥ 90th percentile^c^	≥90th percentile^d^	TG ≥ 110 mg/dl^e^	≥110 mg/dl
			HDL ≤ 40 mg/dl^f^	
Ford et al. [26] 3 out of 5^a^	WC ≥ 90th percentile^g^	≥90th percentile^d^	TG ≥ 110 mg/dl^e^	≥110 mg/dl additional analysis with ≥100 mg/dl
			HDL ≤ 40 mg/dl^f^	
de Ferranti et al. [27] 3 out of 5^a^	WC ≥ 75th percentile	≥90th percentile	TG ≥ 100 mg/dl	≥110 mg/dl
			HDL ≤ 50 mg/dl	

^a^Number of criteria that must be fulfilled for diagnosing MetS.

^b^For Europid males/females; ethnic-specific percentiles are recommended for other population groups [25].

^c^Age- and sex-specific, recommended by NHANES III (National Health and Nutrition Examination Survey).

^d^Age-, sex-, and height-specific, recommended by NHBPEP (National High Blood Pressure Education Program).

^e^Age-specific, recommended by NCEP (National Cholesterol Education Program).

^f^All ages/sexes, recommended by NCEP.

^g^Sex-specific, recommended by NHANES.

The IDF definition differs from the others in several aspects: first, it requires the presence of abdominal obesity as a mandatory condition. Accordingly, only individuals with large WC plus two or more risk factors are diagnosed with MetS [28]. Second, in spite of WC, the remaining four risk factors are determined by using cutoff points that normally apply to adults [28]. In contrast, the other definitions choose lower limit values or use age-, sex-, and high-specific percentiles for determining elevated blood pressure (BP) and dyslipidemia [6, 26, 27]. Third, the IDF suggests that only children above 10 years of age should be examined for MetS, whereas in younger individuals, WC measurement alone should be used for screening [28].

Due to the high number of classifications, comparison across prevalence data obtained from different epidemiological studies in children is difficult [29]. Furthermore, the diagnostic accuracy and predictive value of childhood MetS for future health consequences have not yet been validated [12].

The aim of this literature review is to provide an overview of the current prevalence numbers of pediatric MetS worldwide as well as to discuss the discrepancies between the different definitions and the rationales for this continuing disagreement. Furthermore, the clinical importance of pediatric MetS will also be discussed in order to identify recommendations for its practical application. The following sections analyze the results of several epidemiological studies from the past 5 years that are based on four widely used definitions for pediatric MetS: the definition of the IDF, Cook et al. [6], Ford et al. [26], and de Ferranti et al. [27].

## Search methodology

Primary literature, comprising published journal articles and research papers as well as guidelines, was obtained via Web of Science and PubMed search engines. The chosen articles were sorted according to topic and relevance. In order to find additional literature, reference lists as well as related articles were searched.

The systematic search of the current literature covering the prevalence of pediatric MetS took place in July 2018. The MeSH term “metabolic syndrome”, declared as “major topic”, was combined with MeSH subheadings like: “Definition”, “Epidemiology”, and “Prevalence”. In order to expand the range of results, an additional search query was created by replacing the term “metabolic syndrome” with “insulin resistance”. The search was then specified via defining the exclusion criteria and adding appropriate filters: date of publication within the last 5 years; only articles written in English; articles that covered human research; articles of which the full text was available; and articles that dealt with children (birth—18 years).

Since many different pediatric definitions are used in scientific literature, a goal of this work was to identify the most frequently used ones. Finally, four MetS classifications (IDF, Cook et al., Ford et al., and de Ferranti et al.) were selected and discussed.

Those articles that obtained prevalence data exclusively from adults or from specifically predefined cohorts (e.g., prevalence of MetS in clinical populations or participants with an underlying illness) were excluded.

## Results

The search query “Metabolic Syndrome” AND “epidemiology” offered 397 results, while “Insulin Resistance” AND “epidemiology” resulted in 416 articles. Metabolic Syndrome AND “prevalence” reached 296 hits, “Insulin Resistance” AND “prevalence” reached 58. In total, the Web of Science and PubMed search resulted in 1167 articles.

**Fig. 1:**
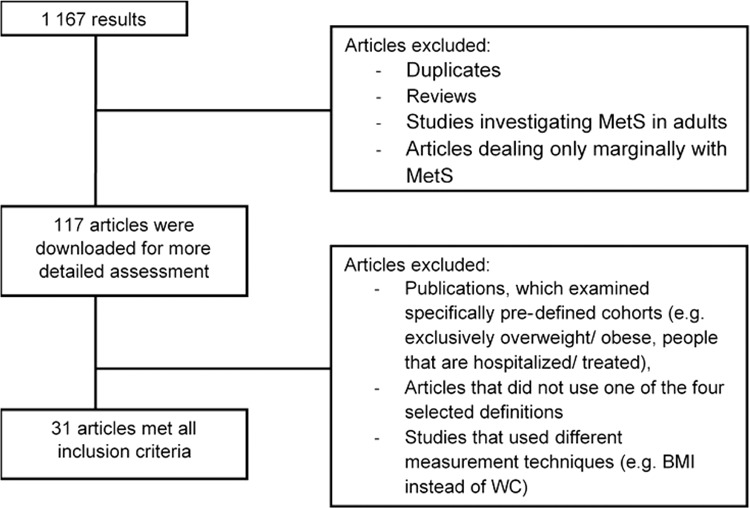
Search methodology flow chart. The panels on the left show the number of publications and the panels on the right show the applied exclusion criteria.

After eliminating the duplicates, reviews or articles in non-Enlish language, 117 papers were downloaded. As the publications differed significantly in terms of their study cohorts and MetS criteria, focus was primarily laid on randomized trials, which were based on the four chosen definitions (IDF, Cook et al., Ford et al., and de Ferranti et al., respectively). In the course of a second evaluation, two cross-sectional studies, in which the recruitment was not randomized, were subsequently not included. However, as they contained relevant information, they are discussed in the latter part of this literature review. Finally, the search resulted in 31 publications, which reflect the prevalence data of the MetS among children and adolescents in different countries worldwide and which were published during the past 5 years (Fig. 1).

Among these, 24 studies used the definition of the IDF, 15 that of Cook et al., eight that of de Ferranti et al., and six studies used the Ford et al. classification. Several publications (*n* = 12) applied more than one definition to the very same cohort in order to compare the differences between the prevalence rates. These results are discussed separately in the section “Comparative Studies.” Furthermore, two longitudinal studies that examined the stability of the MetS status over a follow-up period were analyzed separately in the discussion section.

## Update of the current literature

### The prevalence of MetS among children and adolescents—findings of current scientific review

Table 2 provides an overview of 31 epidemiological studies that met the inclusion criteria. The chosen publications included both large population-based studies as well as smaller surveys, which were conducted in schools or in certain country regions. Sample sizes reached from 371 students in a South African cohort [30] to a maximum of 37,504 adolescents participating in a nationwide study in Brazil [31].

**Table 2:** Epidemiological studies investigating the prevalence of metabolic syndrome in children and adolescents based on the diagnostic criteria from the IDF, Cook et al. [6], Ford et al. [26], and de Ferranti et al. [27].

Author	Year	Country	Study cohort		Study design	IDF criteria	Modified NCEP criteria
			*N*	Age (years)			
Europe							
Ostrihoňová et al. [58]	2017	Slovak Republic	1294	10–17.99	Visitors of Health Advice Centers no randomization	♂2.7% ♀2.8%	
Vanlancker et al. [18]	2017	10 European Countries	1004	12.5–17	Randomized multicenter study not population-representative	2.7%	Cook 3.5%
Galera-martínez et al. [48]	2015	Spain	379	12–16.9	Population-based sample of adolescents living in Almería	3.8%	Ford 5.7%
Ahrens et al. [40]	2014	8 European Countries	12,319	2–10.9	Population-based not nationally representative	0.4%	Cook 1.4%
North America							
Reina et al. [64]	2017	USA	1137	10–16	Population-based study of Latino/Hispanic people living in the US; SOL youth^a^	3.1% (10–15) ♂2.8% (16) ♀6.3% (16)	
Rodríguez et al. [37]	2016	USA	1623	12–19	Nationally representative sample of adolescents; NHANES^b^ 2005–2012	5.4%	
MacPherson et al. [46]	2016	Canada	1228	10–18	Population representative survey; CHMS^c^ 2007–2009, 2009–2011	2.1%	
Miller et al. [20]	2014	USA	3495	12–19	Nationally representative sample of adolescents; NHANES 2001–2010		Ford 10.1%
South America							
Reuter et al. [80]	2018	Brazil	1200	12–17	Population representative sample of adolescents from Southern Brazil	2.1%	Cook 1.9% de Ferranti 5.0%
Ramírez-Vélez et al. [32]	2016	Colombia	1922	9–17.9	Population-based sample of school children in Bogota; FUPRECOL^d^ study 2014–2015	0.3%	Cook 6.2% Ford 7.8% de Ferranti 11.0%
Suarez-Ortegón et al. [50]	2016	Colombia	494	5–9	Cross-sectional study of the scholar population in the city of Cali; IFRECNTEC^e^ study		de Ferranti 8.7%
Kuschnir et al. [31]	2016	Brazil	37,504	12–19	Representative of adolescents from medium- and large-sized cities; ERICA^f^ Study	2.6%	
Burrows et al. [38]	2015	Chile	667	12–17	Adolescents of low to middle SE-status residing in the city of Santiago not population representative	9.5%	
Villalobos Reyes et al. [43]	2014	Venezuela	916	9–18	Representative sample in the city of Mérida; CREDEFAR^g^ Study 2010–2011	1.5%	Cook 2.2%
Dias Pitangueira et al. [41]	2014	Brazil	540	7–14	Random sample from the municipality of Mutuípe, Brazil		de Ferranti12.8%
Agudelo et al. [51]	2014	Colombia	851	10–18	Beneficiaries of a health-promotion company in the city of Medellín no randomization	0.9%	Cook 3.8% Ford 4.1% de Ferranti 11.4%
Asia							
Song et al. [42]	2017	China	831	7–18	“National household-based study in nine Chinese provinces;” CHNS^h^ 2009	1.4% (10–18)	Cook 3.4% (7–18) 3.6% (10–18)
Xu et al. [57]	2017	China	11,174	10–17	Population survey conducted in six provinces in 2007–2011		Cook 3.8%
Lee et al. [60]	2017	South Korea	623	10–18	Population representative; KNHANES^i^	1.0%	
Asghari et al. [33]	2017	Iran	1424	11–18	Randomly selected, population representative; TLGS^j^, baseline 1999	8.4%	Cook 13.1% de Ferranti 26.4%
Bahrani et al. [35]	2016	Iran	538	14–18	Random sampling procedure. 12 high schools in Shiraz, Iran		Cook 6.1%
Kim et al. [52]	2016	Korea	2330	10–18	Population representative; KNHANES 2010–2012	2.1%	Ford 5.7%
Wang et al. [44]	2015	China	1770	7–17	Random sample of 10 schools in urban area of Guangzhou, China	1.1% (10–17)	Cook 2.5% (7–17)
Al-Hussein et al. [36]	2014	Saudi Arabia	2 149	6–17	Random sample of students in Riyadh; S.Ch.O.O.Ls^k^ study	2.0%	Cook and Ford 4.9% de Ferranti 17.5%
Fadzlina et al. [49]	2014	Malaysia	1014	13	Population-based study; students from urban and rural schools	2.6%	
Li et al. [39]	2014	China	910	11–16	Students from 30 high-school classes in North East China	7.6%	
Hosseinpanah et al. [34]	2013	Iran	1424	11–18	Randomized, population representative; TLGS, baseline 1999–2001		Cook 13.3%
Australia							
Huang et al. [81]	2013	Australia	964	17	Representative sample of adolescents from Western Australia	2.7%	
Africa							
Sekokotla et al. [30]	2017	South Africa	371	13–18	Selected high schools in Mthatha, South Africa		Cook ♂6.0% ♀3.1%
Benmohammed et al. [47]	2015	Algeria	989	12–18	Randomized recruitment of school children in the city of Constantine	♂ 1.3% ♀ 0.5%	Cook ♂2.6% ♀0.6% de Ferranti ♂4.0% ♀2.0%
Matsha et al. [45]	2013	South Africa	1272	10–16	Randomly selected from primary schools 2007–2008	1.9%	

^a^Study of Latino Youth.

^b^National Health and Nutrition Examination Survey.

^c^Canadian Health Measures Survey.

^d^In Spanish Associación de la Fuerza Prensil con Manifestaciones de Riesgo Cardiovascular Tempranas en Niños y Adolescentes Colombianos.

^e^Identification of risk factors for adult non-communicable chronic disease in schooled population.

^f^Estudo de Riscos Cardiovasculares em Adolescentes (study of cardiovascular risk in adolescents).

^g^Evaluation of growth, development, and cardio-metabolic risk factors in school children and adolescents from Mérida, Venezuela.

^h^China Health and Nutrition Survey.

^i^Korea National Health and Nutrition Examination Survey.

^j^Teheran Lipid and Glucose Study.

^k^Saudi Children’s Overweight, Obesity and Lifestyles Study.

**Fig. 2:**
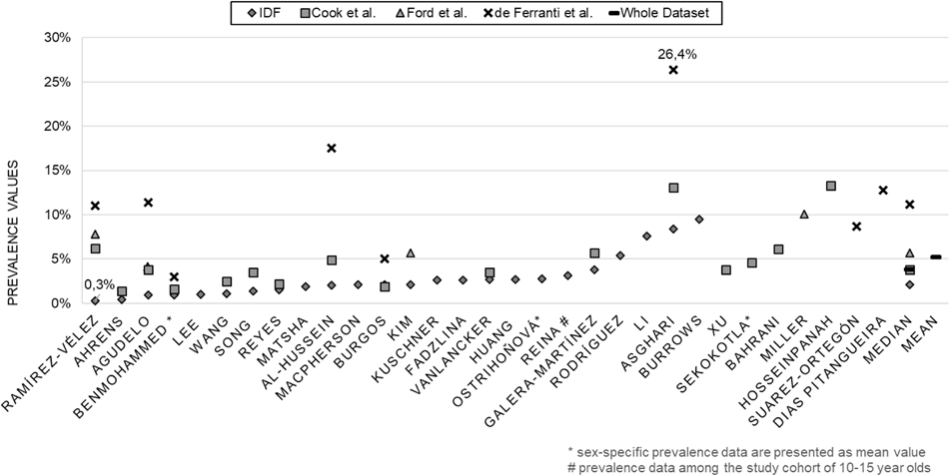
Prevalence of metabolic syndrome based on the definitions from the IDF, Cook et al. [6], Ford et al. [26] and de Ferranti et al. [27]. Minimum: 0.3% in Colombia [32], maximum: 26.4% in Iran [33], mean value of the whole dataset: 5.2%, median value of the whole dataset: 3.8%.

**Fig. 3:**
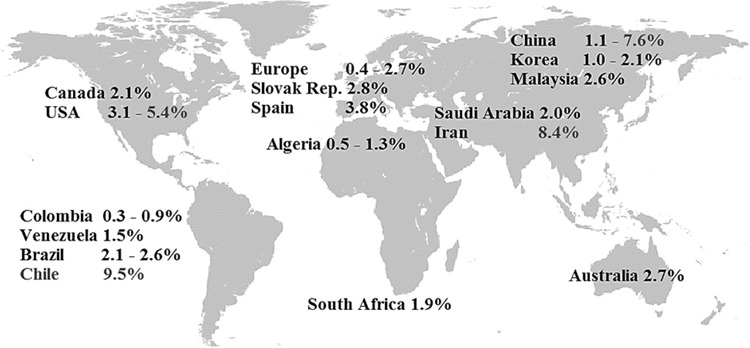
Prevalence of metabolic syndrome in children and adolescents according to the IDF classification. The prevalence is shown as a percentage range and is depicted for selected countries.

**Fig. 4:**
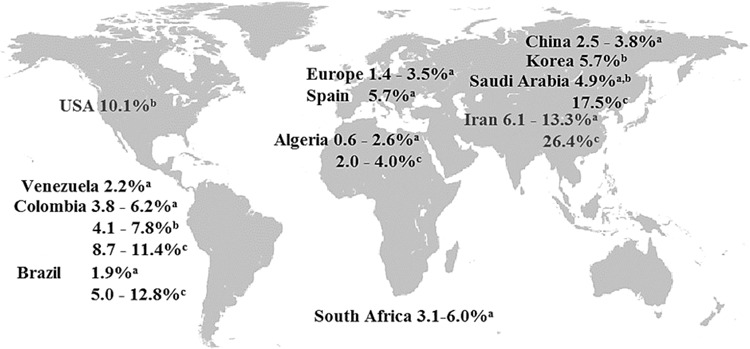
Prevalence of metabolic syndrome in children and adolescents according to the definitions proposed by Cook et al. [6], Ford et al. [26], and de Ferranti et al. [27]. The prevalence is shown as a percentage range and is depicted for selected countries.

Among the 31 included papers, the prevalence of pediatric MetS ranged from 0.3 to 26.4% (see Fig. 2). The lowest prevalence (0.3%) was found in a population-based survey of Colombian school children by using the definition of the IDF [32] (see Figs. 2 and 3). The highest prevalence (26.4%) was found among Iranian children and adolescents by using the definition of de Ferranti et al. (see Figs. 2 and 4) [33]. Prevalence was high in the Middle East (13 and 6% in Iran [33–35] and almost 5% in Saudi Arabia [36] according to the Cook definition), in the United States (5.4% by IDF [37] and 10.1% [20] by the Ford et al. classification), and in South American countries (9.5% in Chile by IDF and 6.2% in Colombia by the Cook definition [32, 38] (see Figs. 3 and 4).

The median calculation of the whole dataset of prevalence numbers yielded 3.8%. Regarding each definition separately, the median values were 2.1% for the IDF, 3.8% for Cook et al., 5.7% for Ford et al., and 11.2% for de Ferranti et al. (see Fig. 2). The mean value of the whole dataset was higher, 5.23%, which is due to the high prevalence levels yielded by de Ferranti´s definition. 21 out of 24-IDF-based results lay within the range between 0.3% [32] and 5.4% [37]. In the remaining three studies, the IDF-based prevalence was considerably higher: 7.6% in China [39], 8.4% in Iran [33], and 9.5% in Chile [38] (Figs. 2 and 3). The modified NCEP criteria, on the other hand, generally resulted in higher prevalence rates, which varied from 1.4 [40] to 26.4% [33] (Figs. 2 and 4).

Although the absolute prevalence of MetS is somewhat low, it is still high considering the young age of the participants and concerning in terms of the potential lifelong burden of disease [11, 20]. Besides, the number of children presenting with at least one risk factor is considerably higher. For example, Miller et al. [20] identified two third (73.2%) of the study population of the “National Health and Nutrition Examination Survey” (NHANES) as having one or more metabolic abnormality, whereas Dias Pitangueira et al. [41] even identified 85.7%. The prevalence rates might seem even more alarming when extrapolated to the total population. According to Song et al. [42], prevalence of 3.4% would mean that more than 11 million Chinese children are affected with MetS and prevalence of 10.1%, found by Miller et al. [20], would mean that 3.3 million US adolescents fulfill the diagnostic criteria. In the majority of included studies central obesity (assessed via WC measurement) and/or dyslipidemia (low HDL and/or high TG) were the most frequent risk factors seen [20, 31, 33, 34, 39, 43–50], whereas high fasting glucose was the least frequent [30, 31, 35, 36, 41–43, 49, 51]. The prevalence of abdominal obesity varies between nations and across ethnic groups: high among European [48], North American [20, 46], and South African [30, 45] study groups in contrast to Asian study populations [52, 53]. Miller et al. reported that in the US study population different ethnicities showed different prevalence of Mets: Hispanic adolescents (14.6%) > non-Hispanic whites (9.8%) > non-Hispanic blacks (5.2%) [20]. These findings are similar to those that were reported previously in US-American NHANES population [6, 19, 27, 37, 53, 54].

There appears to be a direct correlation between BMI and the prevalence of MetS, as MetS is higher among overweight and obese children [31, 32, 34, 35, 37, 39, 41, 43, 47–49, 52]. The prevalence of MetS ranges from 10% [49] up to 57.4% [37] among obese children and adolescents and the prevalence increases with BMI [32, 35, 39, 49, 55, 56].

Some authors report an association between aging and MetS. While this relationship is well established in adults [35, 40, 44, 50, 57–59], it is not so clear in children. Lee and colleagues, for example, observed that the prevalence of MetS was lower in 10–18-year-old children (1.0%) as compared to 19–25-year-olds (2.4%) [60] but other researchers did not observe this association [18, 43, 48, 51], or even reported an inverse correlation [32, 33, 61]. Ramírez-Vélez et al. [32] suggested that the inverse correlation in the younger group could be due to higher prevalence of overweight in this group while Asghari et al. proposed that it could be attributed to pubertal development [33]. Overall, overweight and obesity—rather than age— appear to have greater impact on the development of pediatric MetS [32].

With regard to sex differences, some studies reported that Mets is higher in boys as compared to girls [20, 30, 34, 35, 42–45, 47, 49] but others did not report similar findings [32, 40, 42, 48, 50, 52, 60].

### Comparative studies and discrepancies between the definitions

When evaluating the results from different population-based studies, it is striking that the proportion of children being affected by the MetS varies considerably. This dispersion can be attributed to the properties of the study cohorts such as age-range, sex distribution, ethnicity and prevalence of obesity as well as dietary habits, physical inactivity and environment and socio-economic status [35, 46, 62].

However, when comparing the results from Miller et al. [20]. and Rodríguez et al. [37], it appears that these influencing factors are insufficient in rationalizing such great discordance between prevalence numbers, since both research groups used data from the same nationwide health survey program of the US population (NHANES 2001–2010 and 2005–2012). The most striking difference was that Miller et al. [20] applied the IDF definition, whereas Rodríguez et al. [37] chose the definition of Ford et al. The Ford et al. classification resulted in prevalence nearly twice as high as the IDF classification (10.1% vs. 5.4%). Considering these two definitions differ in their cutoff values and diagnostic requirements (e.g., the IDF determine abdominal obesity as a mandatory diagnostic criterion), it is plausible that they also differ in prevalence outcome. On the other hand, differences between the Miller et al. [20] and Rodriguez et al. [37] data could have arisen due to the different years from NHANES that they included. It is also possible that the prevalence has increased over time in the U.S., which would also cause a higher prevalence rate in the Rodriguez et al. paper [20].

Several research groups compared different classifications in order to explore their degree of discordance (see Table 3). In applying more than one definition to the same study population, these studies could determine an inconsistency in the prevalence outcome: the definition of de Ferranti et al. always reached the highest prevalence while the IDF criteria presented by far the lowest number of children being diagnosed with MetS. Cook et al. and Ford et al. obtained rather similar results (6.2% vs. 7.8% [32], 4.9% vs. 4.9% [36], 3.8% vs. 4.1% [51]) that lay in between de Ferranti’s and the IDF definition.

**Table 3:** Different weighting of components of the metabolic syndrome.

Author (year)	Definition	Diagnostic criterion: elevated BP	Prevalence of elevated BP
Kim et al. (2016) [52]	IDF	Systolic BP ≥ 130 mmHg or diastolic BP ≥ 85 mmHg	2.4%
	Ford	≥90th percentile; specific for age, sex, and high	20.4%
Agudelo et al. (2014) [79]	IDF	Systolic BP ≥ 130 mmHg or diastolic BP ≥ 85 mmHg	2.2%
	Cook	≥90th percentile; specific for age, sex, and high	10.3%
Villalobos et al. (2014) [77]	IDF	Systolic BP ≥ 130 mmHg or diastolic BP ≥ 85 mmHg	0.7%
	Cook	≥90th percentile; specific for age, sex, and high	8.7%

Author year	Definition	Diagnostic criterion: high fasting blood glucose (FBG)	Prevalence of high FBG
Agudelo et al. (2014) [79]	Cook de Ferranti	FBG ≥ 110 mg/dl	0.6%
	IDF, Ford	FBG ≥ 100 mg/dl	2.8%

The results that differed the most came from an Saudi Arabian survey, where the number of children being diagnosed by de Ferranti’s classification as having MetS was eight times higher than by the IDF definition (17.5% vs. 2%). The Ford and Cook classifications yielded nearly 5% [36]. Similar findings were reported by an Iranian study, where, according to the criteria of de Ferranti et al., 26.4% of the study cohort were diagnosed with MetS. The definition of Cook et al. resulted in 13%, whereas the IDF classification identified 8.4% as being affected [33]. In summary, the results from comparative studies demonstrate that the lack of a standardized, globally accepted definition impedes comparison between prevalence rates of different study populations or communities [52].

Another reason for the discrepancies could be attributed to the different weighting of the components of MetS. Although most definitions agree in using the same five components, they differ in terms of the cutoff values. By setting either strict or generous thresholds, individual components of MetS are weighted differently. Strict cutoff values are more likely to be exceeded and increase the number of diagnoses. De Ferranti et al. set the most stringent limits on WC (>75th percentile), HDL (≤50 mg/dl), and TG (≥100 mg/dl) [27, 51]. Therefore, the probability of surpassing these strict thresholds is high. This explains why the definition of de Ferranti et al. results in higher prevalence values than other classifications.

On the other hand, generously set cutoff values are less likely to be surpassed. This means that among the MetS-positive group, only few individuals show this component as being abnormal. Furthermore, higher values (or lower, as in the case of HDL) result in reduced number of people meeting the criteria. For instance, IDF uses adult cutoff points for defining increased BP (systolic BP ≥ 130 mmHg, diastolic BP ≥ 85 mmHg) instead of age-, sex-, and height-specific percentiles [28, 51]. These cutoff values seem too high for children and adolescents and thus might be responsible for the low prevalence by IDF criteria [32]. Findings from Kim and So [52], Agudelo et al. [51], and Reyes et al. [43] confirmed that the prevalence of elevated BP was considerably lower according to IDF criteria than using definitions by Ford et al. or Cook et al. (demonstrated in Table 3). This means that, compared with the remaining components, BP contributes less to the overall number of MetS diagnoses [40].

Similar considerations apply to the definitions of Cook et al. and de Ferranti et al., who chose higher limits for elevated blood sugar (≥110 mg/dl). This way, fewer individuals fulfill the criterion of high fasting glucose. Consequently, blood glucose contributes less to the diagnosis of pediatric MetS than the other components [40].

Strictly defined risk factors determine characteristics of a MetS-positive cohort. Among the four classifications, IDF and Ford et al. use the strictest limit value for fasting blood glucose (>100 mg/dl). Thus, individuals that are at risk of developing T2DM are more likely to be identified by these two criteria than by others (Cook et al. >110 mg/dl). However, Cook et al. and Ford et al. classifications use age-, sex-, and high-specific percentiles for defining elevated BP and thus are assumed to better identify children who are at high risk of developing CVD compared to the IDF classification [51].

### Rationales for the difficulties in developing a uniform MetS definition: current challenges and perspectives

The reasons for the continuing disagreement among criteria and definitions are numerous. Since knowledge about the pathogenic mechanisms of MetS in children and adolescents is insufficient, the development of one reliable definition is problematic [22]. The paucity of long-term studies makes it difficult to quantify the extent to which childhood risk factors actually cause health consequences in adulthood [63, 64]. Therefore, it is challenging to set reliable pediatric threshold values above which the health risk is verifiably increased [64]. Tracking at-risk children into adulthood—with morbidity and/or mortality as end-points—would help determining appropriate cutoff points for young age groups [18, 22, 63, 64].

As the period between childhood and adult age is characterized by huge physiological changes and transition, evaluation of the cardio-metabolic state during this period is extremely difficult and insecure [23]. In the course of growth and puberty, the organism undergoes several modifications in physiological processes that also affect cardio-metabolic parameters [52, 65]. Several factors influence growth during puberty: genetic, nutritional, endocrine, and ethnic [66]. In addition, the onset of puberty is also believed to be associated with a greater risk of development of obesity and CVD in adulthood [67]. Some studies have assessed pubertal growth and cardio-metabolic risk, including measurements of risk factors for CVD and type 2 diabetes [67]. These authors reported an independent association between pubertal timing and adult-MetS-related derangements in more than 5000 subjects (males and females).

Furthermore, the natural rise in insulin levels during puberty is a critical confounding factor and IR has been shown to play a key role in the metabolic changes in obese children [68]. Data from 334 obese 5–19 years old children show that cardio-metabolic risk is associated with increased postprandial IR and dyslipidemia in prepubertal and increased fasting IR in postpubertal obese children [68]. Therefore, cardio-metabolic risk assessment in adolescents requires cutoff points that also reflect pubertal development [69].

Variations in anthropometric and metabolic characteristics between ethnic groups may also hamper development of a uniform definition of MetS [20, 70, 71]. Ethnic-specific cutoff points and percentiles are required to define abdominal obesity, dyslipidemia, elevated BP, and impaired glucose metabolism [28, 43, 45]. Since such percentiles are not available for every population, some studies use preexisting reference values of other population groups instead. This may overestimate or underestimate the actual prevalence of MetS and impedes comparability between different cross-sectional/epidemiological studies [30, 43]. Taken together, cardio-metabolic risk assessment in children and adolescents requires age- and sex-specific percentiles that also consider pubertal development and ethnicity [12, 14, 23, 54, 69, 70, 72].

An additional challenge for a MetS definition is the dichotomous manner of analyzing its components [62]. Each diagnostic criterion is described as either being normal or abnormal as being beneath or above the cutoff values. The definitions do not take into account to what extent an individual risk factor contributes to the overall risk profile [23]. Regarding the whole syndrome, an individual can only be diagnosed as either having MetS or not, whereas the severity of the MetS cannot be evaluated [73]. Therefore, researchers claim that dichotomizing each diagnostic component results in loss of important information. Instead, other research has suggested that measurements should be treated as continuous variables [22, 29], meaning that each risk factor should be involved in the overall risk estimation, regardless of whether it surpasses the threshold or not [18]. For this reason, the continuous MetS has been developed. It is calculated by summarizing the *z*-scores of each MetS component. A high score equates to a poor metabolic profile with higher risk for CVD and T2DM [62]. In this way, the overall cardio-metabolic risk could be evaluated with respect to small/gradual changes of each risk factor and thus better reflect the pathophysiological processes [44]. Overall, more research is needed on whether variables should be treated as continuous or not.

### The relevance of pediatric MetS in clinical practice

Given the continuing disagreement and lack of knowledge regarding pediatric MetS, it is difficult to provide recommendations about its clinical application [74]. Results from longitudinal studies are also not that conclusive. In two Iranian studies childhood MetS was found to be a poor prognostic factor for adult MetS [33, 34] and in the “Cardiovascular Risk of Young Finns Study,” the accuracy in predicting adult health outcomes was hardly better than prediction by pure chance [75]. These findings agreed with the “Princeton Lipid Research Cohort Study,” in which the within-person accordance between childhood- and adult-MetS severity (evaluated by calculation of a MetS severity *z*-score) was found to be moderate [76]. This instability in MetS status could be explained by changes in weight status as well as by pubertal development during the follow-up period [33, 34, 77]. Individuals, who were obese in childhood, but were of normal weight in adulthood, were shown to have a similar risk for being MetS-positive as those who never have been obese [34]. This indicates that weight status per se is a predictor of adult health risk [78]. Indeed, longitudinal studies revealed that childhood MetS could predict adult MetS, T2DM, or CVD, however the predictive value of MetS was not superior to that of BMI per se [34, 78]. These findings foster the discussion whether its clinical application provides any benefit, especially since body weight assessment requires less effort [78].

Despite these multiple doubts, researchers recognize childhood obesity with premature accumulation of cardio-metabolic risk factors as being one major challenge of public health [23, 51, 71, 77]. Indeed, the persistence or worsening of childhood cardio-metabolic risk factors and MetS into adulthood is assumed to substantially increase the risk for future diseases [76, 79]. Therefore, screening measures seem indispensable for identifying those children that are at high risk—especially when considering that initial cardio-metabolic changes do not cause any symptoms [22, 50, 69].

Albeit the utility of MetS in pediatric patients is questionable, its concept of risk factor clustering could serve as a rough guide for risk assessment in the clinical setting [74]. However, instead of sticking to certain MetS classifications, pediatricians should rather focus on established cardio-metabolic risk factors [12, 34, 74, 78]. Since weight status correlates closely with cardio-metabolic abnormalities, WC measurement and BMI calculation are considered helpful screening tools for detecting children with high risk of MetS [39, 47]. Besides this, the individual medical history, familial predisposition, eating habits and lifestyle behavior should also be part of the cardio-metabolic risk evaluation [22, 61, 63]. Considering that MetS already occurs in youth and often in combination with obesity, it is important to start countermeasures as early as possible [51]. Implementing prevention programs against overweight and weight gain in a school environment would reach a large number of children and might realize a decrease in childhood obesity [36]. Such programs should promote knowledge and awareness of a healthy lifestyle with special focus on adapting a healthy diet and boosting physical activity [38]. Treatment strategies should also be targeted at managing obesity as well as treating the individual cluster of cardio-metabolic risk factors [22, 74].

## Limitations

Several limitations have to be mentioned when discussing the results of this literature review. First, not every cohort of the included studies was representative for the overall country population. In some cases, the participant recruitment was conducted in specific country regions [43, 44, 48] or among special ethnic [64] or socio-economic groups [38]. In two studies, the participants were not randomly selected [51, 58]. Second, some studies used data that were collected several years ago [20, 33, 42] and thus might not exactly reflect the current epidemiological situation. Third, due to the exclusion criteria (e.g., studies with particular selection criteria, studies that used different definitions, etc.) prevalence data of several other countries could not be taken into account.

Additional limitations include the broad range of ages included in some studies, which combined children and adolescents (who may be physiologically quite different) as well as the lack of sex-specific estimates in some studies.

## Conclusions and perspectives for future scientific research

Current epidemiological studies revealed that the prevalence numbers of childhood MetS are high in the US, in the Middle East and in South American countries, with the highest proportion of MetS diagnoses occurring among overweight and obese individuals. The high number of different pediatric MetS definitions creates variations in the prevalence data from different epidemiological surveys and impedes comparison between them. Moreover, the actual reliability of the pediatric MetS diagnosis to predict future health consequences is rather poor. This could partly be explained by puberty-associated changes in hormonal status and body weight.

As MetS is on the rise in children and adolescents, and given the disagreement on the diagnosis of MetS in children and youth, cardio-metabolic risk evaluation should rather be based on established risk factors such as nutritional status, hypertension, dyslipidemia, IR, clinical status, and familial predisposition. Future research should take into consideration several aspects:

First, in order to validate the causality between childhood risk factors and adult health consequences, longitudinal studies are required. This will lead to clarification of the underlying pathophysiological mechanisms and facilitate determination of appropriate limit values/ranges [18, 64]. Similarly, the influence of pubertal development on cardio-metabolic parameters needs further investigation [69].

Second, age-, sex-, and ethnicity-specific percentiles for growth, weight, and WC should become available for every population group in each country [71]. This is important as variations in anthropometric and metabolic characteristics between ethnic groups are seen [20, 70, 71]. Specifically, ethnic-specific cutoff points and percentiles are required to define abdominal obesity, dyslipidemia, elevated BP, and impaired glucose metabolism [28, 43, 45]. Using preexisting reference values of other population groups (e.g., Caucasian values for Asian populations) may overestimate or underestimate the actual prevalence of MetS [30, 43].

Last, while there is temptation to suggest one valid, globally accepted definition of MetS for pediatric populations [64], we believe that it is more appropriate to suggest definitions of MetS that are specific to males vs. females, as well as being specific to race/ethnicity or geographic region. Establishment of a global definition of MetS may be too simplistic and will, as this article shows, lead to misinterpretation or underrepresentation of this important cardiovascular risk factor in children and young adolescents.

## References

[1] Li R, Li W, Lun Z, Zhang H, Sun Z, Kanu JS (2016). Prevalence of metabolic syndrome in Mainland China: a meta-analysis of published studies. BMC Public Health.

[2] Bleich SN, Cutler D, Murray C, Adams A (2008). Why is the developed world obese?. Annu Rev Public Health.

[3] Ranasinghe P, Mathangasinghe Y, Jayawardena R, Hills AP, Misra A (2017). Prevalence and trends of metabolic syndrome among adults in the Asia-Pacific region: a systematic review. BMC Public Health.

[4] Ng M, Fleming T, Robinson M, Thomson B, Graetz N, Margono C (2014). Global, regional, and national prevalence of overweight and obesity in children and adults during 1980–2013: a systematic analysis for the Global Burden of Disease Study 2013. Lancet.

[5] Bray GA, Popkin BM (2013). Calorie-sweetened beverages and fructose: what have we learned 10 years later. Pediatr Obes.

[6] Cook S, Weitzman M, Auinger P, Nguyen M, Dietz WH (2003). Prevalence of a metabolic syndrome phenotype in adolescents: findings from the third National Health and Nutrition Examination Survey, 1988–1994. Arch Pediatr Adolesc Med.

[7] Cruz ML, Weigensberg MJ, Huang TT, Ball G, Shaibi GQ, Goran MI (2004). The metabolic syndrome in overweight Hispanic youth and the role of insulin sensitivity. J Clin Endocrinol Metab.

[8] Bastien M, Poirier P, Lemieux I, Després JP (2014). Overview of epidemiology and contribution of obesity to cardiovascular disease. Prog Cardiovasc Dis.

[9] Yumuk V, Tsigos C, Fried M, Schindler K, Busetto L, Micic D (2015). European guidelines for obesity management in adults. Obes Facts.

[10] WHO. Obesity and overweight. 2016. http://who.imt/mediacentre/factsheets/fs311/en/2016.

[11] OECD. Health at a glance 2017. OECD. https://www.oecd.org/social/health-at-a-glance-19991312.htm.

[12] Kassi E, Pervanidou P, Kaltsas G, Chrousos G (2011). Metabolic syndrome: definitions and controversies. BMC Med.

[13] Mendis S, Puska P, Norrving B, World Health Organisation, World Heart Federation, World Stroke Organisation. Global atlas on cardiovascular disease prevention and control. In: Mendis S, et al., editors. Geneva: World Health Organization; 2011.

[14] Kaur J (2014). A comprehensive review on metabolic syndrome. Cardiol Res Pract.

[15] Alberti KG, Eckel RH, Grundy SM, Zimmet PZ, Cleeman JI, Donato KA (2009). Harmonizing the metabolic syndrome: a joint interim statement of the International Diabetes Federation Task Force on Epidemiology and Prevention; National Heart, Lung, and Blood Institute; American Heart Association; World Heart Federation; International Atherosclerosis Society; and International Association for the Study of Obesity. Circulation.

[16] Eckel RH, Grundy SM, Zimmet PZ (2005). The metabolic syndrome. Lancet.

[17] Al-Hamad D, Raman V (2017). Metabolic syndrome in children and adolescents. Transl Pediatr.

[18] Vanlancker T, Schaubroeck E, Vyncke K, Cadenas-Sanchez C, Breidenassel C, González-Gross M (2017). Comparison of definitions for the metabolic syndrome in adolescents. The HELENA study. Eur J Pediatr.

[19] Johnson WD, Kroon JJ, Greenway FL, Bouchard C, Ryan D, Katzmarzyk PT (2009). Prevalence of risk factors for metabolic syndrome in adolescents: National Health and Nutrition Examination Survey (NHANES), 2001–2006. Arch Pediatr Adolesc Med.

[20] Miller JM, Kaylor MB, Johannsson M, Bay C, Churilla JR (2014). Prevalence of metabolic syndrome and individual criterion in US adolescents: 2001-2010 National Health and Nutrition Examination Survey. Metab Syndr Relat Disord.

[21] Friend A, Craig L, Turner S (2013). The prevalence of metabolic syndrome in children: a systematic review of the literature. Metab Syndr Relat Disord.

[22] Steinberger J, Daniels SR, Eckel RH, Hayman L, Lustig RH, McCrindle B (2009). Progress and challenges in metabolic syndrome in children and adolescents: a scientific statement from the American Heart Association Atherosclerosis, Hypertension, and Obesity in the Young Committee of the Council on Cardiovascular Disease in the Young; Council on Cardiovascular Nursing; and Council on Nutrition, Physical Activity, and Metabolism. Circulation.

[23] Mameli C, Zuccotti GV, Carnovale C, Galli E, Nannini P, Cervia D (2017). An update on the assessment and management of metabolic syndrome, a growing medical emergency in paediatric populations. Pharmacol Res.

[24] Ford ES, Li C (2008). Defining the metabolic syndrome in children and adolescents: will the real definition please stand up?. J Pediatr.

[25] Expert Panel on Detection, Evaluation, and Treatment of High Blood Cholesterol in Adults. Executive summary of the third report of The National Cholesterol Education Program (NCEP) expert panel on detection, evaluation, and treatment of high blood cholesterol in adults (adult treatment panel III). JAMA. 2001;285:2486–97. 10.1001/jama.285.19.2486 11368702

[26] Ford ES, Ajani UA, Mokdad AH (2005). The metabolic syndrome and concentrations of C-reactive protein among U.S. youth. Diabetes Care.

[27] de Ferranti SD, Gauvreau K, Ludwig DS, Neufeld EJ, Newburger JW, Rifai N (2004). Prevalence of the metabolic syndrome in American adolescents: findings from the Third National Health and Nutrition Examination Survey. Circulation.

[28] Zimmet P, Alberti KG, Kaufman F, Tajima N, Silink M, Arslanian S (2007). The metabolic syndrome in children and adolescents - an IDF consensus report. Pediatr Diabetes.

[29] Reinehr T, de Sousa G, Toschke AM, Andler W (2007). Comparison of metabolic syndrome prevalence using eight different definitions: a critical approach. Arch Dis Child.

[30] Sekokotla MA, Goswami N, Sewani-Rusike CR, Iputo JE, Nkeh-Chungag BN (2017). Prevalence of metabolic syndrome in adolescents living in Mthatha, South Africa. Ther Clin Risk Manag.

[31] Kuschnir MC, Bloch KV, Szklo M, Klein CH, Barufaldi LA, Abreu Gde A (2016). ERICA: prevalence of metabolic syndrome in Brazilian adolescents. Rev Saude Publica.

[32] Ramírez-Vélez R, Anzola A, Martinez-Torres J, Vivas A, Tordecilla-Sanders A, Prieto-Benavides D (2016). Metabolic syndrome and associated factors in a population-based sample of schoolchildren in Colombia: the FUPRECOL Study. Metab Syndr Relat Disord.

[33] Asghari G, Eftekharzadeh A, Hosseinpanah F, Ghareh S, Mirmiran P, Azizi F (2017). Instability of different adolescent metabolic syndrome definitions tracked into early adulthood metabolic syndrome: Tehran Lipid and Glucose Study (TLGS). Pediatr Diabetes.

[34] Hosseinpanah F, Asghari G, Barzin M, Ghareh S, Azizi F (2013). Adolescence metabolic syndrome or adiposity and early adult metabolic syndrome. J Pediatr.

[35] Bahrani R, Chan YM, Khor GL, Rahman HA, Esmailzadeh A, Wong TW. The relationship between metabolic syndrome and its components with socio-economic status amon adolescents in Shiraz. Southeast Asian J Trop Med Public Health. 2016;47:263–76. 27244965

[36] Al-Hussein FA, Tamimi W, Al Banyan E, Al-Twaijri YA, Tamim H (2014). Cardiometabolic risk among Saudi children and adolescents: Saudi childrens overweight, obesity, and lifestyles (S.Ch.O.O.Ls) study. Ann Saudi Med.

[37] Rodríguez LA, Madsen KA, Cotterman C, Lustig RH (2016). Added sugar intake and metabolic syndrome in US adolescents: cross-sectional analysis of the National Health and Nutrition Examination Survey 2005-2012. Public Health Nutr.

[38] Burrows R, Correa-Burrows P, Reyes M, Blanco E, Albala C, Gahagan S (2016). High cardiometabolic risk in healthy Chilean adolescents: associations with anthropometric, biological and lifestyle factors. Public Health Nutr.

[39] Li P, Jiang R, Li L, Liu C, Yang F, Qiu Y (2014). Prevalence and risk factors of metabolic syndrome in school adolescents of northeast China. J Pediatr Endocrinol Metab.

[40] Ahrens W, Moreno LA, Mårild S, Molnár D, Siani A, De Henauw S (2014). Metabolic syndrome in young children: definitions and results of the IDEFICS study. Int J Obes.

[41] Dias Pitangueira JC, Rodrigues Silva L, Portela de Santana ML, Monteiro da Silva Mda C, de Farias Costa PR, D’Almeida V (2014). Metabolic syndrome and associated factors in children and adolescents of a Brazilian municipality. Nutr Hosp.

[42] Song P, Yu J, Chang X, Wang M, An L. Prevalence and correlates of metabolic syndrome in Chinese children: the China Health and Nutrition Survey. Nutrients. 2017;9:79. 10.3390/nu9010079 PMC5295123 28106792

[43] Reyes M, Mederico M, Paoli M, Briceño Y, Miliani Y, Gómez-Pérez R, et al. Metabolic syndrome in children and adolescents from Mérida city, Venezuela: comparison of results using local and international reference values (CREDEFAR study). Endocrinología y Nutrición. 2014;61:474–85. 10.1016/j.endonu.2014.03.009 24840131

[44] Wang J, Zhu Y, Cai L, Jing J, Chen Y, Mai J (2016). Metabolic syndrome and its associated early-life factors in children and adolescents: a cross-sectional study in Guangzhou, China. Public Health Nutr.

[45] Matsha TE, Kengne AP, Yako YY, Hon GM, Hassan MS, Erasmus RT (2013). Optimal waist-to-height ratio values for cardiometabolic risk screening in an ethnically diverse sample of South African urban and rural school boys and girls. PLoS One.

[46] MacPherson M, de Groh M, Loukine L, Prud’homme D, Dubois L (2016). Prevalence of metabolic syndrome and its risk factors in Canadian children and adolescents: Canadian Health Measures Survey Cycle 1 (2007-2009) and Cycle 2 (2009–2011). Health Promot Chronic Dis Prev Can..

[47] Benmohammed K, Valensi P, Benlatreche M, Nguyen MT, Benmohammed F, Pariès J (2015). Anthropometric markers for detection of the metabolic syndrome in adolescents. Diabetes Metab.

[48] Galera-Martínez R, García-García E, Vazquez-Lopez M, Ortiz-Pérez M, Ruiz-Sánchez A, Martín-González M (2015). Prevalence of metabolic syndrome among adolescents in a city in the Mediterranean area: comparison of two definitions. Nutr Hosp.

[49] Fadzlina AA, Harun F, Nurul Haniza MY, Al Sadat N, Murray L, Cantwell MM (2014). Metabolic syndrome among 13 year old adolescents: prevalence and risk factors. BMC Public Health.

[50] Suarez-Ortegón MF, Aguilar-de Plata C (2016). Prevalence of metabolic syndrome in children aged 5–9 years from southwest colombia: a cross-sectional study. World J Pediatr.

[51] Agudelo GM, Bedoya G, Estrada A, Patiño FA, Muñoz AM, Velásquez CM (2014). Variations in the prevalence of metabolic syndrome in adolescents according to different criteria used for diagnosis: which definition should be chosen for this age group?. Metab Syndr Relat Disord.

[52] Kim S, So WY. Prevalence of metabolic syndrome among Korean adolescents according to the National Cholesterol Education Program, adult treatment panel III and International Diabetes Federation. Nutrients. 2016;8:588. 10.3390/nu8100588 PMC5083976 27706073

[53] Duncan GE, Li SM, Zhou X-H (2004). Prevalence and trends of a metabolic syndrome phenotype among U.S. adolescents, 1999–2000. Diabetes Care.

[54] Dong B, Arnold L, Peng Y, Wang Z (2016). Ethnic differences in cardiometabolic risk among adolescents across the waist-height ratio spectrum: National Health and Nutrition Examination Surveys (NHANES). Int J Cardiol.

[55] Figueroa Sobrero A, Evangelista P, Kovalskys I, Digón P, López S, Scaiola E (2016). Cardio-metabolic risk factors in Argentine children. A comparative study. Diabetes Metab Syndr..

[56] Ataie-Jafari A, Heshmat R, Kelishadi R, Ardalan G, Mahmoudarabi M, Rezapoor A (2014). Generalized or abdominal obesity: which one better identifies cardiometabolic risk factors among children and adolescents? The CASPIAN III Study. J Trop Pediatr.

[57] Xu T, Liu J, Liu J, Zhu G, Han S (2017). Relation between metabolic syndrome and body compositions among Chinese adolescents and adults from a large-scale population survey. BMC Public Health.

[58] Ostrihoňová T, Rimárová K, Bérešová J, Kontrošová S, Dorko E, Diabelková J (2017). Prevalence and trends of metabolic syndrome in clients of health advice centres during the years 2003–2012. Cent Eur J Public Health.

[59] Rosini N, Moura SA, Rosini RD, Machado MJ, Silva EL (2015). Metabolic syndrome and importance of associated variables in children and adolescents in Guabiruba—SC, Brazil. Arq Bras Cardiol.

[60] Lee K (2017). Metabolic syndrome in Korean adolescents and young adult offspring and their parents. Asia Pac J Clin Nutr.

[61] Monzani A, Rapa A, Fuiano N, Diddi G, Prodam F, Bellone S (2014). Metabolic syndrome is strictly associated with parental obesity beginning from childhood. Clin Endocrinol.

[62] Owens S, Galloway R (2014). Childhood obesity and the metabolic syndrome. Current Atheroscler Rep.

[63] Brambilla P, Lissau I, Flodmark CE, Moreno LA, Widhalm K, Wabitsch M (2007). Metabolic risk-factor clustering estimation in children: to draw a line across pediatric metabolic syndrome. Int J Obes.

[64] Reina SA, Llabre MM, Vidot DC, Isasi CR, Perreira K, Carnethon M (2017). Metabolic syndrome in hispanic youth: results from the Hispanic Community Children’s Health Study/Study of Latino Youth. Metab Syndr Relat Disord.

[65] Reinehr T, Wolters B, Knop C, Lass N, Holl RW (2015). Strong effect of pubertal status on metabolic health in obese children: a longitudinal study. J Clin Endocrinol Metab.

[66] Soliman A, De Sanctis V, Elalaily R, Bedair S (2014). Advances in pubertal growth and factors influencing it: can we increase pubertal growth?. Indian J Endocrinol Metab.

[67] Widén E, Silventoinen K, Sovio U, Ripatti S, Cousminer DL, Hartikainen AL (2012). Pubertal timing and growth influences cardiometabolic risk factors in adult males and females. Diabetes Care.

[68] Tobisch B, Blatniczky L, Barkai L (2015). Cardiometabolic risk factors and insulin resistance in obese children and adolescents: relation to puberty. Pediatr Obes.

[69] Reinehr T (2016). Metabolic syndrome in children and adolescents: a critical approach considering the interaction between pubertal stage and insulin resistance. Curr Diab Rep.

[70] Alberti KG, Zimmet P, Shaw J (2006). Metabolic syndrome-a new world-wide definition. A consensus statement from the International Diabetes Federation. Diabet Med.

[71] Agirbasli M, Tanrikulu AM, Berenson GS (2016). Metabolic syndrome: bridging the gap from childhood to adulthood. Cardiovasc Ther.

[72] Copeland KC, Zeitler P, Geffner M, Guandalini C, Higgins J, Hirst K (2011). Characteristics of adolescents and youth with recent-onset type 2 diabetes: the TODAY cohort at baseline. J Clin Endocrinol Metab.

[73] Lee A, Gurka M, Deboer M. Trends in metabolic syndrome severity and lifestyle factors among adolescents. Pediatrics. 2016;137:e20153177. 10.1542/peds.2015-3177 PMC4771130 26908664

[74] Magge SN, Goodman E, Armstrong SC. The metabolic syndrome in children and adolescents: shifting the focus to cardiometabolic risk factor clustering. Pediatrics. 2017;140:e20171603. 10.1542/peds.2017-1603 28739653

[75] Magnussen CG, Cheriyan S, Sabin MA, Juonala M, Koskinen J, Thomson R (2016). Continuous and dichotomous metabolic syndrome definitions in youth predict adult type 2 diabetes and carotid artery intima media thickness: the cardiovascular risk in Young Finns Study. J Pediatr.

[76] DeBoer MD, Gurka MJ, Woo JG, Morrison JA (2015). Severity of the metabolic syndrome as a predictor of type 2 diabetes between childhood and adulthood: the Princeton Lipid Research Cohort Study. Diabetologia.

[77] Stanley TL, Chen ML, Goodman E (2014). The typology of metabolic syndrome in the transition to adulthood. J Clin Endocrinol Metab.

[78] Koskinen J, Magnussen CG, Sinaiko A, Woo J, Urbina E, Jacobs DR, Jr, et al. Childhood age and associations between childhood metabolic syndrome and adult risk for metabolic syndrome, type 2 diabetes mellitus and carotid intima media thickness: The International Childhood Cardiovascular Cohort Consortium. J Am Heart Assoc. 2017;6:e005632. 10.1161/JAHA.117.005632 PMC5586423 28862940

[79] Morandi A, Maffeis C (2014). Predictors of metabolic risk in childhood obesity. Horm Res Paediatr.

[80] Reuter CP, Burgos MS, Barbian CD, Renner JDP, Franke SIR, de Mello ED. Comparison between different criteria for metabolic syndrome inschoolchildren from southern Brazil. Eur J Pediatr. 2018;177:1471–7. 10.1007/s00431-018-3202-2. Epub 2018 Jul 4. PMID: 29974212. 10.1007/s00431-018-3202-2 29974212

[81] Huang RC, Beilin LJ, Ayonrinde O, Mori TA, Olynyk JK, Burrows S, et al. Importance of cardiometabolic risk factors in the association between nonalcoholic fatty liver disease and arterial stiffness in adolescents. Hepatology. 2013;58:1306–14. 10.1002/hep.26495. Epub 2013 Aug 6. PMID: 23703776. 10.1002/hep.26495 23703776

